# Diagnosis of diastolic dysfunction in the emergency department: really at reach for minimally trained sonologists? A call for a wise approach to heart failure with preserved ejection fraction diagnosis in the ER

**DOI:** 10.1186/s13089-018-0107-2

**Published:** 2018-10-08

**Authors:** Gabriele Via, Guido Tavazzi

**Affiliations:** 1 Cardiac Anesthesia and Intensive Care, Cardiocentro Ticino, Via Tesserete 48, Lugano, Switzerland; 2 Emergency Department, Anaesthesia and Intensive Care Unit, Fondazione IRCCS Policlinico S. Matteo, Pavia, Italy; 3 Department of Clinical, Surgical, Diagnostic and Paediatric Sciences, Anaesthesia, Intensive Care and Pain Therapy Unit, University of Pavia, Pavia, Italy

**Keywords:** Heart failure, Diastolic dysfunction, Heart failure with preserved ejection fraction, Echocardiography, Focused cardiac ultrasound, Lung ultrasound

In developed countries heart failure represents a heavy epidemiological burden, with a prevalence of 1–2% of the adult population, rising to ≥ 10% among people with > 70 years of age [1, 2]. Diastolic heart failure (Heart Failure with preserved Ejection Fraction, HFpEF [2]) accounts for a relevant proportion of all HF admissions, ranging from 22 to 70% according to its definition, setting, population age and sex, with the highest prevalence in the elderly. Furthermore, among people > 65 years of age presenting to primary care with breathlessness on exertion, one in six will have unrecognized HF (mainly HFpEF) [3]. In patients with HFpEF, there is a strong association between prognosis and the underlying heart failure etiology, but overall mortality is estimated as high as 5–10% [4]. These data overall compel an accurate and early diagnostic strategy for HFpEF since its presentation in the emergency department, and early bedside ultrasound undoubtedly has the potential to comply with this need [5].

Preliminary data on the feasibility of a proposed simplified approach to diagnose diastolic dysfunction, “more suitable for the use by emergency physicians (EPs) with limited experience in echocardiography”, have been recently reported on the Critical Ultrasound Journal [6]. Although  the authors address an area of great interest in clinical practice, the work appears flawed by a series of relevant limitations, and altogether fraught by the drawbacks of oversimplification of a complex matter. These flaws touch the key points of bedside diastolic dysfunction assessment and are worth being addressed:

1. HFpEF demands a multi-parametric echocardiographic assessment. In this retrospective analysis of data from a previous observational study on stable hypertensive patients, Del Rios et al. considered a single echocardiographic variable in order to assess concordance of EPs and cardiologists in the diagnosis of diastolic dysfunction: medial/lateral averaged mitral annular tissue doppler (TDI) protodiastolic velocity. Other authors have recently investigated the possibility of diagnosing HFpEF by EPs receiving a dedicated training in cardiac bedside ultrasound [7], or suggested an approach to the issue in the form of concept paper [8]. Others have proposed a simplified approach to diastolic dysfunction diagnosis in specific critical care populations [9]. All these authors, consistently with the known complexity of this diagnosis and in line with current echocardiographic recommendations, have tackled the issue by combining several ultrasound-derived indices. More recently Johansen et al. have demonstrated in a community-based study that a combination of three relatively easy to obtain parameters (e’, E/e’ and indexed left atrial volume) was able to stratify the population for increasing risk of cardiac major adverse events [10]. The last *Recommendations for the Evaluation of Left Ventricular Diastolic Function by Echocardiography* are in fact explicit in warning that none of the echocardiographic indices should be used in isolation, as some measurements may fall in the normal range despite the presence of diastolic dysfunction due to the several hemodynamic factors that may affect each signal [11].
2. TDI acquisition can be technically challenging. Althought E/E’ is key to assess diastolic dysfunction with good reproducibility and reliability, a series of technical issues can affect the accurate measurement of mitral annular TDI. Protodiastolic mitral annular velocity (TDI E’), the cornerstone of diastolic dysfunction diagnosis, reflects mitral annulus motion that precedes filling; it correlates well with invasive measures of the time constant of myocardial relaxation *tau* although it is not entirely governed by relaxation [12]. There are a number of pathological conditions that impair myocardial relaxation and restoration forces with increased lengthening load (LA pressure), resulting in reduced and delayed longitudinal motion and E’ velocity. TDI E’ measurement mandates optimal 2D images to be adequately sampled, being thus affected by gain and filter adjustment, sufficient visualization of mitral annulus, absence of mitral annulus severe calcifications/prosthesis, correct angle (< 20°) of insolation, and adjustment according to the plane of cardiac motion [13, 14]. All these technicalities, with their inherent operator-dependency and learning curve, place this kind of echocardiographic assessment beyond the reach of a limited ultrasound training.
3. TDI interpretation conceals several pitfalls that should not be neglected. There are a variety of pathological conditions where TDI E’ could be normal in presence of altered cardiac dynamics: constrictive physiology [15], patients with moderate to severe primary MR and normal LV relaxation due to increased flow across the regurgitant valve [16], discordance between lateral and septal E’ in advanced systolic heart failure [17], atrial fibrillation, ventricular dys-synchrony or, moreover, regional wall motion abnormalities [13]. None of these conditions are addressed in simplified approaches, such as the one proposed by Del Rios et al.
4. Limited bedside ultrasound diagnostic tests are accurate in specific patient populations, and only when interpreted in tight conjunction with clinical data. One of the basic assumptions of Focused Cardiac UltraSound (FOCUS) (and point of care ultrasound in general) is that the limited ultrasound exam is sufficiently informative only when it is part of a clinical-ultrasound integrated approach [18, 19], not as standalone diagnostic test (e.g. only in shocked patients the absence of severe right ventricular failure allows to rule out pulmonary embolism as a cause). In other words, the limitations of the simplified approach are “compensated” by the magnitude of the ultrasound abnormality (gross finding, easier to detect) and by the specific clinical picture (high pre-test probability). In this respect, a cohort of patients without symptoms of congestive heart failure does not seem to be particularly suitable to test the (even preliminary) hypothesis that diastolic dysfunction may fall within the purview of FoCUS.
5. Core of HFpEF diagnosis is the demonstration of elevated left atrial pressures. Patients may exhibit diastolic dysfunction (as detected by a reduced TDI mitral annular E’) unrelated to their respiratory symptoms, i.e. without having high left atrial pressures and congestive HF. Rather than diastolic dysfunction per se, what matters in the approach to patients with suspected acute heart failure is the finding of positive echocardiographic indices of increased left atrial pressures [13]. These should be indices sufficiently validated in decompensated/critical patients, (such as E/E’, pulmonary veins systolic fraction, E/A, DTE), and are usually assessed with a semi-quantitative approach [20].

The application of echocardiography in the assessment of diastolic function and cardiovascular pathophysiology requires advanced competences and skills (compared to FoCUS) to both acquire high-quality pictures and signals and to interpret them in the context of the acute cardiac care setting. A recent meta-analysis showed how despite the application of the guidelines for the diagnosis of diastolic dysfunction [14] this diagnosis is subject to a high inter-operator variability (from 12 to 84%) [21]. Not surprisingly, unanimous consent drove evidence-based expert opinion not to include diastolic dysfunction among the targets of the FoCUS exam [18, 22–25].

**Fig. 1:**
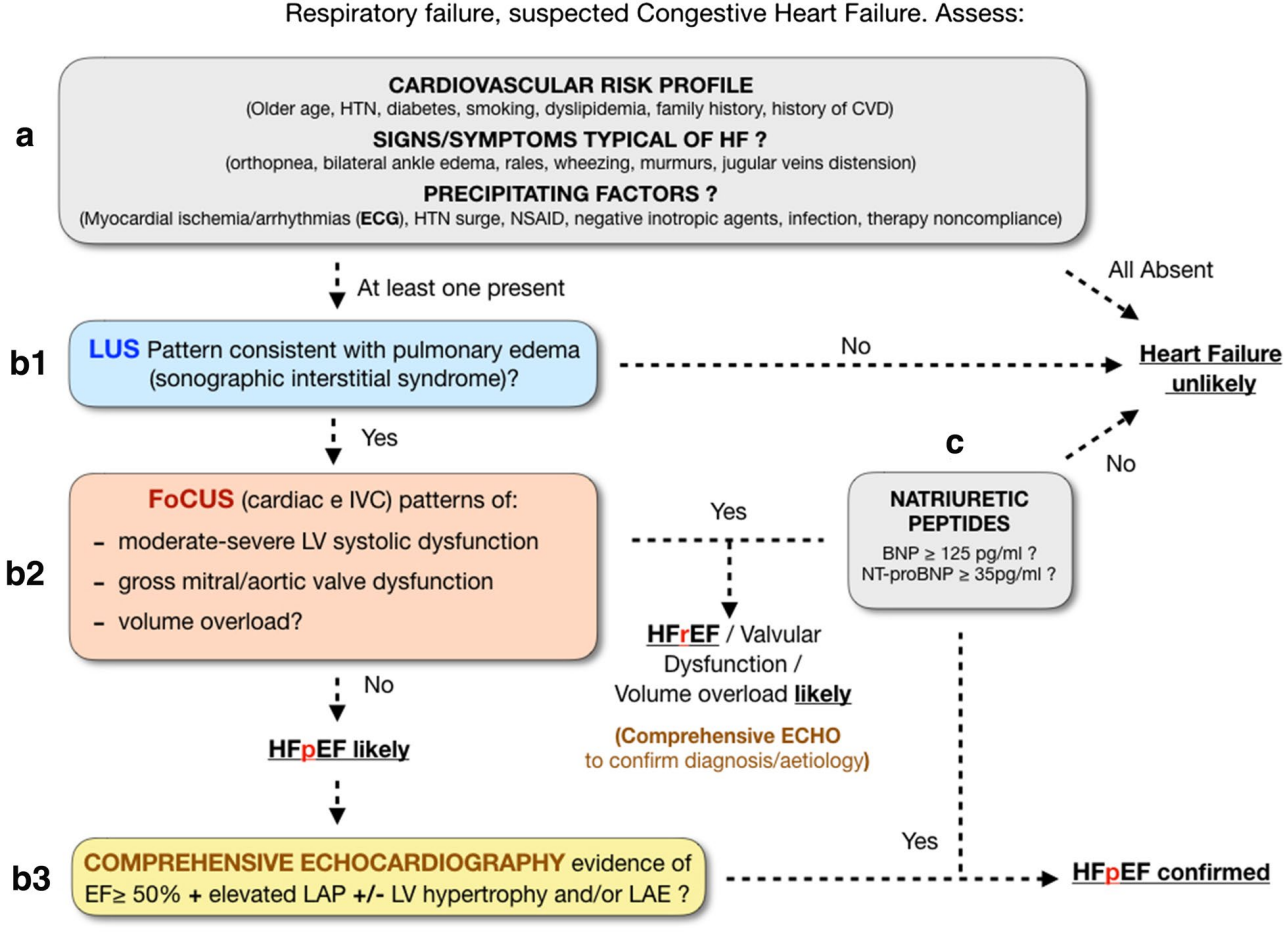
Clinical-ultrasound integrated diagnostic approach to the patient with suspected congestive heart failure, focused on the diagnosis of heart failure (HF) with preserved ejection fraction (HFpEF). When facing respiratory distress, and congestive heart failure is suspected, history and a brief clinical exam should screen for cardiovascular risk factors, typical signs/symptoms of heart failure and for potential precipitating factors (step A). Multi-organ bedside ultrasound follows (step B), starting from lung ultrasound (B1): a pattern of bilateral, symmetrical, homogeneously diffuse scans with multiple B-lines (more than 2 positive chest areas per side = sonographic interstitial syndrome) [32] is diagnostic for pulmonary edema. A potential cardiac etiology of this pulmonary congestion is then screened for with Focused Cardiac Ultrasound (B2): the finding of moderate-severe left ventricular systolic dysfunction raises high suspicion for congestive high failure with reduced ejection fraction (HFrEF). This can then be confirmed with the echocardiographic demonstration of high left atrial filling pressures. Alternatively, FoCUS findings of gross valvular dysfunction or pure volume overload (if consistent with history) will suggest a different cardiogenic or a hydrostatic cause of the pulmonary edema. Immediate or delayed comprehensive echocardiography will again confirm the diagnosis and clarify the mechanism and degree of valvular dysfunction. When all the FoCUS findings consistent with potential causes of pulmonary edema are ruled out, comprehensive echocardiography (B3) is even more required to confirm/rule out a likely diagnosis of heart failure with preserved ejection fraction (HFpEF, defined as ejection fraction ≥ 50%, with TDI and Doppler indices diagnostic for elevated left atrial pressures, with/without left ventricular hypertrophy or left atrial enlargement [2]). The diagnosis of HFpEF finally requires a concomitant positive natriuretic peptides assay (step C). Natriuretic peptides must in any case be pathological for the diagnosis of any alternative cardiogenic cause of pulmonary edema (either HFrEF or HF caused by valvular dysfunction). *HTN* hypertension, *CVD* cardiovascular disease, *HF* heart failure, *ECG* electrocardiography, *NSAIDS* non steroid anti-inflammatory drugs, *LUS* lung ultrasound, *FoCUS* focused cardiac ultrasound, *IVC* inferior vena cava, *LV* left ventricle, *EF* ejection fraction, *LAP* left atrial pressure, *LAE* left atrial enlargement, *HFpEF* heart failure with preserved ejection fraction, *HFrEF* heart failure with reduced ejection fraction, *BNP* brain natriuretic peptide

The inherent limitations of FoCUS in the diagnosis of congestive heart failure in the ER can though be partly circumvented by means of clinically integrated multi-organ ultrasound [18, 26, 27]. There is recent evidence that the combination of E/E’ > 15 and lung ultrasound findings consistent with pulmonary congestion has 100% sensitivity and 95.8% specificity in the diagnosis of congestive heart failure, regardless of the ejection fraction [28]. A sequential, systematic, ultrasound approach is in the end appropriate to diagnose HFpEF and should be based on a sound pathophysiological ground (Fig. 1, steps B1, B2, B3): (1) Diagnosing pulmonary congestion (with lung ultrasound) [29, 30], and ruling out other causes of respiratory failure (with multi organ bedside ultrasound) [31, 32]. (2) Ruling out left ventricular systolic dysfunction, gross aortic/mitral valve abnormalities, pure volume overload as potential causes of pulmonary edema (with FoCUS) [18]. (3) Confirming the cardiogenic nature of pulmonary congestion by detecting high left atrial pressures in the absence of reduced ejection fraction and, possibly, structural cardiac abnormalities (comprehensive echocardiography) [2]. In essence, once suspicion of HFpEF is raised, FoCUS should always prompt a confirmatory comprehensive echocardiographic exam. This all should be integrated into the clinical and biochemical standard diagnostic workup (see the clinical-ultrasound integrated approach described in Fig. 1).

One of the pivotal rules in medicine is that the difficult path to diagnosis requires a process of integrative, systematic, acquisition of signs, symptoms and data. This is even more true when a complex operator-dependent tool is applied in critical scenarios. Echocardiography is extremely powerful in the study of cardiovascular pathophysiology, and any message supporting the concept of simplifying a complex diagnosis by studying a single echocardiographic parameter risks to be definitively simplistic and misleading, rather than just hazardous. A comprehensive approach which integrates clinical, biochemical, FoCUS, lung ultrasound, and echocardiographic findings is advised to better approach the suspected diagnosis of diastolic dysfunction causing congestive heart failure.
